# Highly Sensitive Room-Temperature Graphene-Modulated AlGaN/GaN HEMT THz Detector Architecture

**DOI:** 10.3390/s26031006

**Published:** 2026-02-03

**Authors:** Rudrarup Sengupta, Gabby Sarusi

**Affiliations:** Department of Photonics and Electro-Optics Engineering, School of Electrical and Computer Engineering, Ben-Gurion University of the Negev, Beer Sheva 8410501, Israel; sarusiga@bgu.ac.il

**Keywords:** THz detector, AlGaN/GaN HEMT, monolayer graphene, room-temperature THz detection

## Abstract

This work proposes new architecture, supported by analytical modelling and computer-aided design (CAD) simulations, for a highly sensitive monolayer graphene-gated AlGaN/GaN HEMT terahertz (THz) detector operating at room temperature (RT). The monolayer graphene gate acts as a surface plasmon absorber for the incident THz radiation. The carrier density perturbation caused by incident THz energy on the monolayer graphene surface is then capacitively coupled to the two-dimensional electron gas (2DEG) channel of the HEMT structure underneath. The channel is partially depleted for increased mobility and nonlinearity with potential asymmetry across the channel for consistent photogeneration. The Drude absorption of THz radiation initiates intraband transitions in monolayer graphene, thereby reducing phonon losses. These reduced phonon losses enable RT THz detection. Based on our simulations, the proposed detector architecture can generate a responsivity of 2.12 × 10^6^ V/W at 1 THz with a broadband bandwidth of 2 THz.

## 1. Introduction

Sensing the terahertz (THz) to far-infrared (IR) spectral range has baffled researchers in the last twenty years [1,2,3]. Most effective THz sensors need bandgap-engineered structures [4,5]. The biggest impediment to the effective detection of THz signals is their strikingly low photonic energy (4.1 meV for a frequency of 1 THz), as they are fully immersed in thermal noise for any semiconductor even at cryogenic temperature and definitely at room temperature [1,2]. Semiconductors with such small bandgaps (semi-metals) need to operate at very low temperature to overcome the signal-to-noise ratio barrier that is needed for classical photonic detectors [6]. In order to overcome this limitation, for room-temperature operation, THz detectors need to operate not as photonic detectors but as receiving antennas where charge carriers should follow the THz oscillation frequency [7]. Here, the fundamental limitation in detecting THz radiation is the requirement of high electron mobility and the overall high RC constant of the antenna’s equivalent circuit that limits THz oscillation detection [8]. Most THz detection technologies operating at room temperature revolve around the coupling/absorbance of the THz energy in plasmons, typically constrained in some potential wells [7,9]. The interaction of low-energy THz radiation with surface plasmon polaritons to impose photoconductivity is the basic idea behind most successful THz detection and emission strategies.

A sensitive THz detector operating at room temperature needs to successfully convert the low-energy THz radiation absorbed by surface plasmons into detectible photovoltage without the need for cryogenic cooling [7]. Field effect transistors (FETs) can provide a mobile channel for the detection of THz radiation if operated under proper non-resonant conditions [10]. THz detection is possible due to the nonlinear properties of the transistor, which leads to the rectification of an ac current induced by the incoming THz radiation [9,11]. Some potential asymmetry (bias voltage) between the source and drain induces the nonlinear properties in the transistor so that the incoming radiation superimposes ac voltage with a detectible amplitude between the source and the gate. As a result, a rectified photo-response appears in the form of dc voltage between the source and drain which is proportional to the radiation power (photovoltaic effect) [9,10,11]. The most suitable transistors that can track the high frequency required for efficient THz detection are AlGaN/GaN high-electron-mobility transistors (HEMTs). We are interested in the confined triangular quantum well two-dimensional electron gas (2DEG) characteristics of this heterostructure. GaN-based HEMTs have a high 2DEG channel density (~10^13^ cm^−2^) along with low ON-state channel resistance (~100 mΩ/mm) and appreciable Hall mobility (~1700 cm^2^ V^−1^ s^−1^) owing to their strong spontaneous and piezoelectric polarization (~3 MV/cm), large saturation velocity, high breakdown voltage, and ability to operate at room to high temperatures, compared to Si- or GaAs-based HEMTs [12]. The nonlinearity in the 2DEG channel of a transistor has been already proposed for broadband non-resonant detection from millimetre-wave radiation to THz radiation, with the advantages of room-temperature operation, high responsivity, and low noise-equivalent power (NEP) [13]. Non-resonant detection in GaN HEMTs was first demonstrated at 200 GHz [13]. Panasonic Corporation reported a detection responsivity of 1.1 kV/W at 1 THz using a GaN HEMT with 80 nm gate dipole antennas [14]. The detection of 0.9 THz radiation with 3.6 kV/W responsivity and 40 pW/Hz^1/2^ NEP was also achieved in GaN HEMTs using floating antennas [15].

It was found that graphene acts as an excellent surface plasmon absorber of THz radiation, which is technologically quite familiar to 2DEG THz plasmonic physics [16]. Graphene is naturally an excellent material for broadband electrooptic modulators in the infrared, due to its property of controlling the absorption coefficient by interband transitions [16]. In the far-infrared and THz spectral range, intraband transitions dominate, where graphene acts as a Drude absorber [16]. A graphene-based THz FET modulator concept can then be easily implemented by substituting the metallic gate with a graphene layer [17]. Furthermore, a graphene gate would also decrease the attenuation of the incoming THz radiation with respect to a metal one. Overall, modulation depths close to 100% have been predicted for monolayer graphene in the THz frequency range [18].

In this work, we present a detailed simulation-based architecture of an AlGaN/GaN HEMT THz detector with a monolayer graphene gate, which aims to multiply the strong plasmonic effects of graphene and the highly mobile 2DEG of AlGaN/GaN HEMT to create an ultra-sensitive THz detector that can operate at room temperature (RT). Monolayer graphene is used as the surface plasmon absorber of the incident THz radiation, which is then capacity-coupled to the 2DEG channel of the AlGaN/GaN HEMT underneath. The nonlinearity in the 2DEG is maintained for photogeneration. The effective capacitive coupling of the modulating charge carriers (upon THz photon absorption by intraband transitions) to the HEMT’s 2DEG should detect and amplify THz signals. The Drude absorption of low-energy THz radiation by thick metal layers incurs significant losses due to lattice vibrations. Intraband transitions of monolayer graphene can be highly beneficial here, allowing the detector to operate at room temperature, where thermally generated carriers can be manifested as Drude plasmons. The utilization of monolayer graphene as the absorber layer should also reduce phonon losses; hence, THz-induced intraband transitions in graphene should enhance absorptivity, thereby enhancing carrier modulation amplitude. This increased amplitude of oscillations should significantly improve THz responsivity. This simulation-based validation work on our proposed THz detector is an important step to carry out before the actual fabrication and deployment of the detector.

## 2. THz Detector Architecture

**Figure 1:**
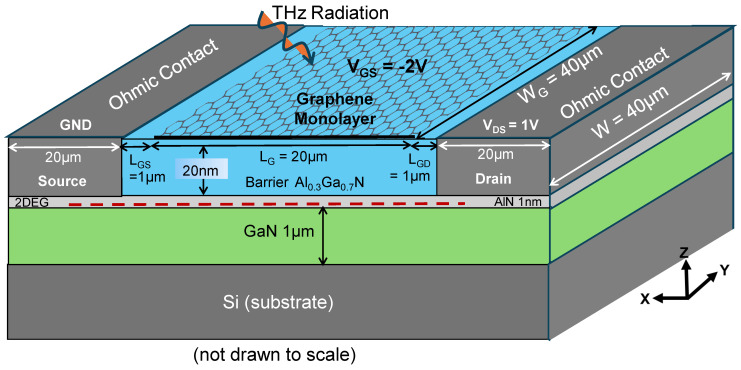
A 3D schematic model of the monolayer graphene-modulated AlGaN/GaN HEMT RT THz detector (not drawn to scale). All transistor dimensions and applied voltages required for proper photogeneration are depicted in the 3D model. The thickness of the Si substrate is taken to be 525 µm, the standard thickness for a 4-inch wafer. Abbreviations: GND—ground; L_G_—gate length; L_GS_—distance between source and gate; L_GD_—distance between gate and drain; W_G_/W—gate width. The red dashed line referrers to the 2DEG in the AlGaN/GaN heterointerface.

In Figure 1, we show a 3D schematic model of the proposed room-temperature THz detector (not drawn to scale). AlGaN/GaN HEMT aids broadband non-resonant detection by coupling the low-energy THz radiation absorbed by monolayer graphene to the highly mobile 2DEG (closely analogous to a superconductor at room temperature), thereby providing a modulating drain current (I_ds_) across the channel. Moreover, the AlGaN(20 nm)/GaN heterostructure is transparent to THz and can be grown effectively on silicon without compromising its mobility [12]. The Drude absorption of THz radiation on the monolayer graphene gate creates wavelength-limited carrier density oscillations, which add up, as a small-signal ac component, to the gate voltage (V_GS_) already applied, without suffering from nearly any phonon losses thanks to monolayer graphene. Therefore, low phonon losses should enable RT THz detection. The capacity coupling of the resultant V_GS_ should induce perturbations in the 2DEG channel beneath the graphene gate. An applied potential gradient across the 2DEG (from source to drain) should then create the required potential non-uniformity/asymmetry. Since the lattice vibration frequency of the strained 2DEG is also in the THz spectral range [10], this is one of the main reasons for the asymmetry. This asymmetry creates the phase difference between the THz-induced polarized electric field and the drift velocity established due to the applied V_DS_. Over the length of the channel, these 2DEG electrons drifting towards the drain suffer from interference due to asymmetry in phase between the electric field and drift velocity. This interference should then rectify the signal, leading to a photogenerated voltage as described by Dyakonov and Shur (D-S) [10]. The combination of capacity-coupled graphene plasmons excited by THz and 2DEG in the AlGaN/GaN HEMT should generate a substantial rectified voltage (ΔU) at room temperature with fast response times.

Based on this concept, we modelled our AlGaN/GaN HEMT detector. The HEMT was modelled according to the architecture shown in Figure 1. For practical purposes, we purposefully avoided including any scattering effects in the HEMT because we are operating the HEMT in partially depleted conditions. Since we intend to operate our HEMT under the lowest field–highest mobility conditions, we gave special attention to accurately calculating the low-field I_ds_. This modelling helps us to affirm our concept that with the highly mobile 2DEG, we can work in the linear region to couple it with the incumbent THz radiation and measure the residual V_DS_ and I_ds_. To design our research and validate the above-mentioned THz detector model, we performed analytical modelling of the AlGaN/GaN HEMT, given in detail in Supplementary Material Sections S1 and S2. These calculations gave us the HEMT’s modelling parameters, as shown in Figure 1.

**Figure 2:**
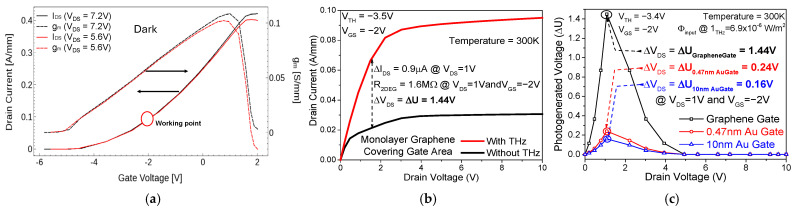
(**a**) Reduced 2DEG concentration (for V_GS_ = −2 V) to perfectly tailor it with the magnitude of incoming THz energy radiation. (**b**) I_ds_-V_ds_ at V_gs_ = −2 V, showing the direct effect of THz rectification with increased potential asymmetry under depleted conditions. Since ΔR_2DEG_ << R_2DEG_, dark R_2DEG_ is shown. (**c**) The THz photogeneration response of the transistor, showing the advantage of using monolayer graphene over a gold gate.

The mechanism of nonlinearity says that the most important effect is the modulation of the electron concentration in the channel and hence of the channel resistance using the local ac gate-to-channel biassing. Because of this, in the expression for the electric current j=env, both the carriers’ charged concentration (n) and their drift velocity (v) will be modulated at the THz radiation frequency. As a result, a dc current will appear as follows: $j_{dc}=e\langle n_{1}(t)v_{1}(t)\rangle$, where $n_{1}(t)$ and $v_{1}(t)$ are the modulated components of n and v, and the angular brackets denote averaging over the oscillation period 2π/ω, with ω being the angular frequency. Under open-circuit conditions, a compensating dc electric field will arise, resulting in the photoinduced source–drain voltage ∆U. Under partially depleted channel conditions, carrier modulation (modulation due to electron density multiplied by the modulation due to drift velocity) is subdued to a particular limit due to the lack of availability of carriers, thereby increasing mobility. Hence the output current appears to be incremented by a dc value, which is the photogenerated current. To find suitable partially depleted channel conditions, we simulated the HEMT using Sentaurus TCAD at different gate voltages, incorporating a relevant nonlinear plasma wave dynamic model. Using the transfer characteristics (I_ds_ vs. V_gs_) of the HEMT, we determined the accurate reduced 2DEG concentration so that when THz is coupled to the 2DEG, the created asymmetry is not overpowered by the high 2DEG concentration, leading to poor ∆U. This is achieved by applying a negative voltage at the gate, lower in magnitude than the threshold voltage (V_th_). The voltage threshold V_th_ of our HEMT model is calculated to be −3.4 V. Figure 2a demonstrates that our working point is at V_gs_ = −2 V, where the 2DEG is only partially depleted. In addition, we applied a drain-to-source voltage V_ds_ = 1 V to maintain the potential asymmetry.

A 3D schematic representation of graphene on the AlGaN/GaN HEMT THz detector upon exposure to THz radiation is given in Supplementary Material Section S3. The EM wave physics simulator in Sentaurus TCAD was used for the virtual THz photogeneration on the detector. The operation of the detector under partially depleted conditions ensures a steady photogenerated voltage. The transistor characteristics (I_ds_-V_ds_) at V_gs_ = −2 V in Figure 2b with and without THz illumination show the direct effect of THz-induced rectification with increased potential asymmetry under depleted conditions. For the simulation of graphene, we used a carbon layer thinned down to a single atomic layer with appropriate optical parameters (n, k values). This is a good approximation for obtaining the Drude carrier modulations of graphene when exposed to THz radiation. With the above-mentioned specifications, we obtain a ΔI_DS_ (photogenerated dark I_DS_) value of 0.9 µA and a photogenerated voltage of 1.44 V at V_ds_ = 1 V. Contrastingly, recent work on the analytical modelling of a graphene gate FET THz detector (with lower channel mobility than HEMT) estimated its photogenerated voltage up to the range of milli-volts [19]. Operation at higher drain voltage is avoided since it incurs significant noise in the detector, which overshadows the photogeneration effect. The input THz flux (Φ_input_) value is taken as 6.857 × 10^−6^ W/m^2^ [20] (area of 20 µm × 40 µm).

To demonstrate the advantage of using monolayer graphene instead of metal at the gate, we simulated the same AlGaN/GaN HEMT structure with a 10 nm Au gate instead of graphene. In many applications, it has been shown that thicker metal gates sometimes act as a faraday cage for incoming electromagnetic radiation [21]. To avoid this, we simulated a monolayer Au gate as well with a thickness of 0.47 nm. The photogeneration results of both the simulations are compared to our monolayer graphene gate detector. The simulations show that with a 10 nm thick Au gate, we get a ΔI_DS_ value of 0.1 µA and a ΔU of only 0.16 V. With the 0.47 nm Au gate, we get a ΔI_DS_ value of 0.15 µA, and ΔU=0.24 V. Comparing these results numerically to our graphene gate THz detector proves that our proposed detector is 6 times better than that with the 0.47 nm Au gate and 9 times better than that with the 10 nm Au gate in terms of ΔU only due to the monolayer graphene gate. This is because a single atomic layer of Au demonstrates lower mobility than monolayer graphene. Figure 2c shows these results graphically.

## 3. Simulated Detector Parameters

**Figure 3:**
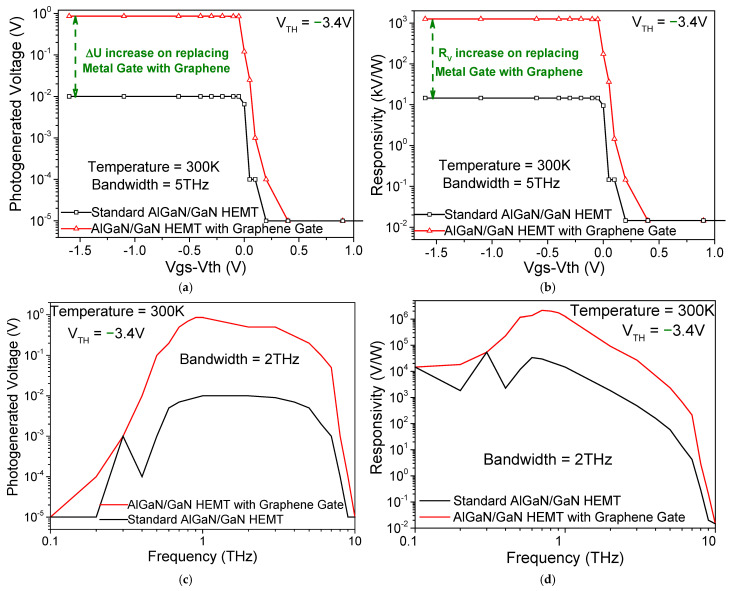
(**a**) Photogenerated voltage and (**b**) responsivity with respect to gate voltage swing are plotted and compared for AlGaN/GaN HEMT detectors with monolayer graphene gate and Au gate (standard AlGaN/GaN HEMT). (**c**) Photogenerated voltage and (**d**) responsivity with respect to incident radiation frequency are plotted and compared for AlGaN/GaN HEMT detectors with monolayer graphene gate and Au gate (standard AlGaN/GaN HEMT).

Responsivity is calculated by computing the average ΔU (ΔU=1.44 V) per unit input flux. Based on the simulation, we found that such a detector may provide a record-high value of responsivity (R_v_) of 2.12 × 10^6^ V/W. Figure 3 shows a detailed analysis of photogenerated voltage and R_v_ with respect to gate voltage swing (V_gs_-V_th_) and photogenerated light frequency. We included a comparative analysis of the detector parameters for a monolayer graphene gate and an identical HEMT transistor with a Au metal gate (denoted as standard AlGaN/GaN HEMT in Figure 3). The simulation results predict quite promising detector parameters. We can observe a two-order magnitude increase in photogenerated voltage and responsivity at 1 THz in a partially depleted 2DEG channel for the monolayer graphene gate, compared to the Au metal gate at room temperature, in Figure 3a and Figure 3b, respectively. Simulations of the same ΔU for various frequencies between 0.1 THz and 10 THz demonstrate the broadband detection mechanism of the HEMT due to D-S instability and asymmetry-influenced rectification. We achieved a bandwidth (BW) of 2 THz with ΔU and R_v_ peaking at 1 THz. Because of negligible phonon losses during THz transmission by monolayer graphene, we can achieve such high photogeneration and responsivity with our monolayer graphene gate compared to a standard Au metal gate HEMT THz detector.

The combination of the Drude absorption of incident THz radiation (with resultant intraband transitions) by the monolayer graphene gate, which is capacity-coupled to the partially depleted high-mobility 2DEG channel of the AlGaN/GaN HEMT, and nonlinear 2DEG channel behaviour due to the application of potential asymmetry enables the elimination of any antenna/grating structures. Compared to conventional D-S photodetector architecture [11], our detector can achieve photogeneration without any antenna structure due to the negligible phonon losses of the absorbing monolayer graphene gate at room temperature (since the entire gate absorber layer is made of single-atomic-layer graphene) and the tailored potential asymmetry provided in the channel. A near-perfect partial depletion of the channel ensured just the right carrier concentration of the 2DEG for the best photogeneration. The partially depleted channel considerably increases the mobility of the carriers, and this increase in mobility becomes extremely crucial for room-temperature THz photodetectors. For example, ΔU drops down to below 0.7 V with gate voltages close to zero. Moreover, in the THz regime, the real part (direct, in-phase response of currents) of graphene conductivity dominates compared to the imaginary part (inductive, out-of-phase response), which also helps in the effective Drude absorption and consequent photogeneration of incident THz radiation [22].

**Figure 4:**
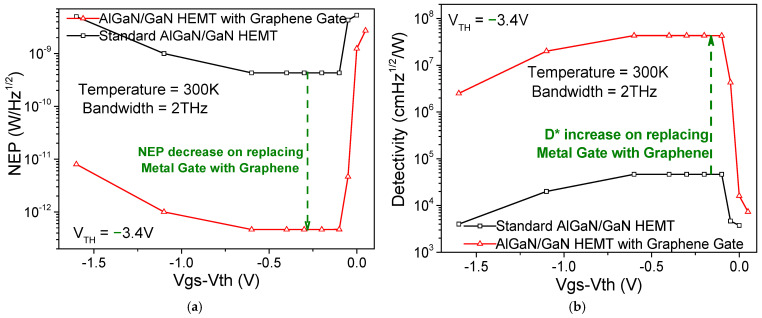
(**a**) NEP and (**b**) detectivity are plotted with respect to gate voltage swing and compared for AlGaN/GaN HEMT detectors with monolayer graphene gate and Au gate (standard AlGaN/GaN HEMT).

To calculate the NEP and detectivity (D*) of our modelled detector, standard noise factors like quantum noise, background noise, and detector noise are considered. General values for the background noise at room temperature (300 K) are taken as a reference for all our calculations. The final step of analytical calculations gave us NEP and detectivity values which are comparable to some of the best infrared bolometric detectors [23]. Our calculations gave us NEP = 4.64 × 10^−12^ W/√Hz, SNR = 110.71 dB, and D* = 4.31 × 10^7^ cm√Hz/W. We compare NEP and D* here as well for our monolayer graphene gate and an identical HEMT transistor with a Au metal gate in Figure 4a and Figure 4b, respectively. We can observe a three-order decrease in NEP and an analogous three-order increase in D* due to negligible phonon losses by using a monolayer graphene gate.

Another important parameter required to measure the quality of the detector is the dynamic range (DR). It is defined as the ratio of the largest non-saturating input signal (photocurrent) i_max_ to the smallest detectable input signal i_min_. The largest non-saturating signal is given by $i_{max}=\frac{qQ_{max}}{t_{int}}$, where q is the electronic charge, $Q_{max}$ is the effective well capacity, and $t_{int}$ is the integration time (20 ps). $DR=\frac{qQ_{max}/t_{int}}{Noise}$. Our calculations gave DR = 81.59 dB. Moreover, we compared our newly designed THz detector with one of the best-performing RT THz detectors designed with gold nano-pillars on the gate [24], which also has peak responsivity at 1 THz and a BW of 2 THz. A detailed comparative chart with all the detector parameters is given in Table 1. It is to be noted that ours is a simulation-based work, whereas the study with the THz detector designed with gold nano-pillars on the gate [24] is an experimental work. Nano-pillar-gated detectors are very popular for intensifying THz photogeneration, but our introduction of monolayer graphene enables capturing the entire incident THz energy with negligible losses. This is why our proposed detector shows a 3 times improvement in ΔU, a 1.5-order improvement in R_v_, 2 orders of improvement in NEP, and a 1-order improvement in D* compared to the nano-pillar gate detector. We give a more generalized comparative analysis of the detector parameters of different genres of THz detector technologies with respect to our proposed technology in Supplementary Material Section S4. In Supplementary Material Section S5, we provide a detailed comparative table of the operating frequency, responsivity, and active area dimensions in previous works which use dimensions in a micrometre range similar to ours, which proves the effectiveness and basis of the dimensions of the detector that we selected. For practical purposes, a plurality of our proposed detector model will be placed in a focal plane array for efficient THz photogeneration.

## 4. Conclusions

In this work we demonstrated the use of a simulation-based model of an RT THz detector with a monolayer graphene-gated AlGaN/GaN HEMT. The monolayer graphene gate acts as a Drude absorber of the incident THz radiation. The Drude absorption of THz radiation on the graphene gate creates wavelength-limited carrier density oscillations, which add up, as a small-signal ac component, to the applied gate voltage, without suffering from nearly any phonon losses due to the single-atomic-layer structure of graphene, which enables RT THz detection. The resultant gate voltage is then capacity-coupled with the highly mobile 2DEG channel of the AlGaN/GaN HEMT inducing perturbations. A negative voltage is applied on the gate to partially deplete the 2DEG channel, thereby suppressing the intrinsic carrier modulation and increasing mobility. An applied potential gradient across the 2DEG (V_DS_) creates the required potential nonlinearity which in turn creates a phase difference between the THz-induced polarized electric field and drift velocity, and this creates interference due to asymmetry in phase between the electric field and drift velocity. Hence, the output current/voltage is increased by a dc value, which is defined as the photogenerated ΔU. Our graphene-modulated AlGaN/GaN HEMT RT THz architecture shows an R_V_ of 2.12 × 10^6^ V/W, NEP of 4.64 × 10^−12^ W/√Hz, SNR of 110.71 dB, D* of 4.31 × 10^7^ cm√Hz/W, and DR = 81.59 dB, which are some of the best detector parameters predicted to date. We utilized a perfectly tuned channel with proper nonlinearity conditions to optimally utilize monolayer graphene’s Drude absorber capabilities in THz, without utilizing any grating gate, and this is a unique feature of our work. This simulation-based validation work conducted on our proposed THz detector is an important step before the actual fabrication and deployment of the detector. Since the parameters are calculated from a simulation-based model, upon fabrication, the detector parameters might degrade slightly.

## Figures and Tables

**Table 1:** Comparison of detector parameters of our graphene on GaN HEMT THz detector and one of best THz detectors [24].

Models	Residual Voltage (ΔU)	Responsivity (R_v_)	NEP (BW = 2 THz)	Detectivity (BW = 2 THz)
**Graphene on AlGaN/GaN HEMT**	**1.44 V**	**2.12 × 10^6^ V/W**	**4.64 × 10^−12^ W/√Hz**	**4.31 × 10^7^ cm√Hz/W**
Nano-Pillar Gate AlGaN/GaN HEMT [24]	0.5 V	1.45 × 10^5^ V/W	4.30 × 10^−9^ W/√Hz	2.09 × 10^5^ cm√Hz/W

## Data Availability

The original contributions presented in this study are included in the article/Supplementary Materials. Further inquiries can be directed to the corresponding author.

## Supplementary Materials

The following supporting information can be downloaded at https://www.mdpi.com/article/10.3390/s26031006/s1. AlGaN/GaN HEMT modelling (Section S1). Drude modelling of monolayer graphene (Section S2). Working principle with 3D illustration of our THz detector (Section S3). Comparative analysis table of detector parameters (Section S4). Comparative analysis table of detectors with similar dimensions (Section S5).
