# Supplementary material for: Highly Sensitive Room-Temperature Graphene-Modulated AlGaN/GaN HEMT THz Detector Architecture
Source: Sensors (Basel). 2026 Feb 3;26(3):1006. doi: 10.3390/s26031006 (PMC12900085; doi:10.3390/s26031006)
Supplement: Supplementary file 1 [file sensors-26-01006-s001.zip › sensors-4038859-supplementary.pdf]

Highly Sensitive Graphene-Modulated AlGaN/GaN HEMT Room-Temperature THz Detector Architecture

Supporting Information

Rudrarup Sengupta* and Gabby Sarusi

Department of Photonics and Electro-Optics Engineering, School of Electrical and Computer Engineering, Ben-Gurion University of the Negev, Beer Sheva, Israel.

*Correspondence author, Orcid ID: 0000-0002-0670-918X; email: rudrarup@post.bgu.ac.il

1: AlGaN/GaN HEMT modelling

To design our research and validate our THz detector model, we performed analytical modelling of AlGaN/GaN HEMT to determine threshold voltage and drain current characteristics. For GaN/AlGaN/GaN HEMT structures, the sheet electron concentration $n_s(x)$ can be calculated by using the total bound sheet charge $\sigma(x)$ and the following equation[1],

$$n_s(x) = \left\{1 + \frac{\varepsilon(x)}{\varepsilon(0)}\frac{d_{GaN}}{d_{AlGaN}}\right\}^{-1}\left\{\frac{\sigma_x}{e} - \left(\frac{\varepsilon_0\varepsilon(x)}{d_{AlGaN}e^2}\right) \times \left[e\Phi_b^{eff}(x) + E_F(x) - \Delta E_c\right]\right\} \quad (1)$$

where, $\varepsilon(x)$ is the relative dielectric constant of $Al_xGa_{1-x}N$, d_{AlGaN} and d_{GaN} are the thicknesses of the barrier and the cap layer, $e\Phi_b^{eff}(x)$ is the effective Schottky barriers of the gate contact on top of AlGaN, $E_F(x)$ is the Fermi level with respect to the GaN conduction-band-edge energy, and ΔE_c is the conduction band offset at the AlGaN/GaN interface where a 2DEG forms. For undoped HEMT structures and assuming that the background concentration of free carriers can be neglected. But in our calculations, we have taken a moderate doping concentration of $10^{18}cm^{-3}$. It is worth noting that our calculations have strictly catered to the tensor values for AlGaN/GaN grown on Silicon than conventional sapphire or SiC. The calculations have rendered an average sheet carrier concentration of 1.83×10^{14} cm^{-2} for d_{AlGaN} = 10nm and 2.89×10^{14} cm^{-2} for d_{AlGaN} = 30nm.

The major challenge in calculating surface potential in HEMT devices is the complicated variation of the fermi level (E_F) with applied biases. A self-consistent solution of the Schrodinger and Poisson's equations in the quantum well, assuming triangular profile for potential is expressed as[2],

$$n_s = DV_{th}\left\{\ln\left(1 + e^{\frac{E_F - E_0}{V_{th}}}\right) + \ln\left(1 + e^{\frac{E_F - E_1}{V_{th}}}\right)\right\} E_{0,1} = \gamma_{0,1}n_s^{2/3} \;; n_s = \frac{\varepsilon}{qd}(V_{go} - E_F - V_x) \quad (2)$$

Where, $V_{go} = V_g - V_{off}$, and V_x is the channel potential at any point x in the channel. V_{off} is the voltage when the channel is fully depleted, and V_g is the gate voltage, hence $V_{off} << V_{th}$, and D is the density of states. In this step we calculate V_{th} for a given d_{AlGaN} using n_s values calculated from equation (2). Detailed analytical

calculations have enabled us to accurately calculate V_{th} values for different d_{AlGaN} values as shown in figure S1. Hence, from figure S1 we can observe around 10% reduction in V_{th} if we thin down the AlGaN layer from 30nm to 20nm but the reduction in effective sheet carrier concentration is negligible (average sheet carrier concentration is of the same order for d_{AlGaN} = 20nm and d_{AlGaN} = 30nm). This interesting tradeoff enables us to set our AlGaN thickness to 20nm and effective threshold voltage V_{th}=-3.4V. A description of all other symbols to be used for further analysis are given below.

$$k_{0,1} = \gamma_{0,1}\left(\frac{C_g}{q}\right)^{\frac{2}{3}}; \qquad V_{gef} = V_g - V_{off} - E_F; \qquad \xi_{0,1} = \exp\left(\frac{E_f - k_{0,1} - V_{gef}^{\frac{2}{3}}}{V_{th}}\right);$$

$$p = \frac{C_g}{q}V_{gef} - \sum_{i=0}^{1} DV_{th}\ln(\xi_i + 1); \qquad q = \frac{C_g}{q} - \sum_{i=0}^{1}\frac{D}{1+\xi_i^{-1}}\left(1 + \left(\frac{2}{3}\right)k_i V_{gef}^{-\frac{1}{3}}\right);$$

$$r = \sum_{i=0}^{1}\frac{(2/9)V_{gef}^{-\frac{4}{3}}Dk_i(1+\xi_i^{-1}) + \frac{D}{V_{th}}\left(1+\frac{2}{3}k_i V_{gef}^{-\frac{1}{3}}\right)^2}{(1+\xi_i^{-1})^2}; \qquad (3)$$

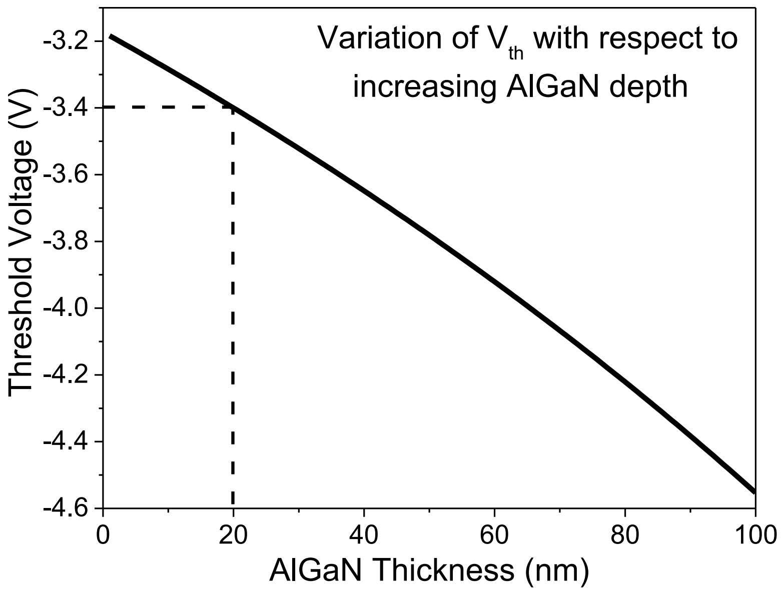

Figure S1. Graph demonstrating variation of V_{th} with respect to increasing AlGaN thickness.

Equation (2) is transcendental in nature and cannot be solved analytically. To obtain E_F and n_s, we have divided the variation of E_F with respect to V_g into three different regions,

1) V_g<V_{off} and the sub-V_{off} region where E_F is comparable to V_{go},
2) V_g>V_{off} and E_F<E_0, demonstrating the moderate 2DEG region,
3) V_g>V_{off} and E_F>E_0, demonstrating a strong 2DEG region.

As in our conditions, the n_s is quite large, we are already working in the strong 2DEG region. We intend to use the HEMT as a linear device, with minimum drain field to bring the field effect mobility close to the Hall mobility values. We want to keep the gate field in deep inversion regime, to maintain a high n_s. This will

aid to couple the weak THz fields and induce significant perturbations in the 2DEG. Regional expressions for $V_g > V_{off}$ can be combined to obtain a single expression for E_F as,

$$E_F = V_{go}\left(1 - H(V_{go})\right) \tag{4}$$

where,

$$H(V_{go}) = \frac{V_{go} + V_{th}[1 - \ln(\beta V_{go}) - \frac{\gamma_0}{3}\left(\frac{C_g V_{go}}{q}\right)^{2/3}}{V_{go}\left(1 + \frac{V_{th}}{V_{go}}\right) + \frac{2\gamma_0}{3}\left(\frac{C_g V_{go}}{q}\right)^{2/3}} \quad and \quad \beta = \frac{C_g}{qDV_{th}} \tag{5}$$

Using equation (2), for $V_x = 0$, we can calculate the expression for E_F in the sub-V_{off} region as,

$$E_{F,sub-V_{off}} = V_{go} - \frac{2qDV_{th}}{C_g} e^{V_{go}/V_{th}} \tag{6}$$

For the compact model, a single unified expression for E_F is desired. Combining equations (4) and (5), we obtain a single unified continuous expression valid for all regions,

$$E_{F,unified} = V_{go} - \frac{2V_{th}\ln\left(1 + e^{-V_{go}/2V_{th}}\right)}{1/H(V_{go}) + \left(C_g/qD\right)e^{-V_{go}/2V_{th}}} \tag{7}$$

These computation steps follow the Householder's steps for solving implicit functions. Hence the surface potential can be calculated as $\Psi = E_f + V_x$ at both ends of the channel.

The first step towards the drain current modelling is the accurate calculation of the critical voltage, $V_{critical}$, which will define the linear and saturation regimes of the HEMT. As we strictly adhere to the linear regime, for THz detection, the $V_{critical}$ calculation becomes important. The critical voltage is given as[3],

$$V_{critical} = \Phi_m(x) - \frac{qN_D}{2\varepsilon(x)}\left(d_{AlGaN} - \frac{n_s}{N_D}\right) \tag{8}$$

where, $\Phi_m(x)$ is the Al concentration dependent Schottly barrier height at the AlGaN/GaN interface, N_D is the intentional AlGaN doping concentration. The drain current in the 2DEG channel can be obtained from the current density equation as,

$$I_{ds} = Wq\mu(y)\left(n_s(y)\frac{dV_c(y)}{dy} + \frac{k_b T}{q}\frac{dn_s(y)}{dy}\right) \tag{9}$$

where W is the gate width, $V_c(y)$ is the channel potential at a position y and $\mu(y)$ is the field dependent mobility given as[4],

$$\mu(y) = \frac{\mu_0}{1 + \frac{1}{E_1}\frac{dV_C(y)}{dy}} \tag{10}$$

with $E_1 = {E_c v_{sat}}/{\mu_0}\, E_c - v_{sat}$, E_c is the saturation electric field, μ_0 is the low field mobility which can be approximated as the 2DEG Hall mobility, and v_{sat} is the saturation drift velocity. Using the equations (9) and (10), the drain current is expressed as,

$$I_{ds} = \frac{Wq\mu_0}{1 + \frac{1}{E_1}\frac{dV_C(y)}{dy}}\left(n_s(y)\frac{dV_C(y)}{dy} + \frac{k_b T}{q}\frac{dn_s(y)}{dy}\right) \tag{11}$$

Using equations (2) and (11), we obtain,

$$I_{ds}\left(1+\frac{1}{E_1}\frac{dV_c(y)}{dy}\right)=\left(\frac{2DWq^2\mu_0\varepsilon(x)}{2Dq^2(d_{AlGaN}+d_{AlN})+\varepsilon(x)}\right)\left(V_g-V_c(y)-V_{th}(x)-\frac{k_bT}{q}\right)\frac{dV_c(y)}{dy} \quad (12)$$

Integrating equation (12) along channel length L, we obtain,

$$I_{ds}=\frac{-\alpha_2+\sqrt{\alpha_2^2-4\alpha_1\alpha_3}}{2\alpha_1}$$

Where,

$$\alpha_1=\left(\frac{2DWq^2\mu_0\varepsilon(x)}{2Dq^2(d_{AlGaN}+d_{AlN})+\varepsilon(x)}\right)(R_{drain}^2-2R_{source}R_{drain})-\left(\frac{2R_{source}R_{drain}}{E_1}\right)\alpha_2$$

$$=L+\frac{V_{ds}}{E_1}+\left(\frac{2DWq^2\mu_0\varepsilon(x)((2R_{source}R_{drain})\left(V_g-V_{th}(x)-\frac{k_bT}{q}\right)-V_{ds}(R_{source}R_{drain})}{2Dq^2(d_{AlGaN}+d_{AlN})+\varepsilon(x)}\right)$$

$$\alpha_3=\left(\frac{2DWq^2\mu_0\varepsilon(x)}{2Dq^2(d_{AlGaN}+d_{AlN})+\varepsilon(x)}\right)\left(\frac{V_{ds}^2}{2}-V_{ds}\left(V_g-V_{th}(x)-\frac{k_bT}{q}\right)\right) \quad (13)$$

R_{source} and R_{drain} are source and drain resistances respectively, and D is the density of states.

The saturation current in the strong inversion regime can be obtained from equation 13 as,

$$I_{dsat}=\left(\frac{2DWq^2\mu_0\varepsilon(x)E_c}{2Dq^2(d_{AlGaN}+d_{AlN})+\varepsilon(x)}\right)\left(V_g-V_{dsat}-V_{th}(x)-\frac{k_bT}{q}\right) \quad (14)$$

where V_{dsat} is the saturation drain voltage calculated by equating the equations (13) and (14) under saturation conditions as,

$$V_{dsat}=\frac{-\beta_2+\sqrt{\beta_2^2-4\beta_1\beta_3}}{2\beta_1}$$

where,

$$\beta_1=\left(\frac{4DWq^2\alpha_1\mu_0\varepsilon(x)E_c}{2Dq^2(d_{AlGaN}+d_{AlN})+\varepsilon(x)}\right)^2\left(1+\frac{R_{source}+R_{drain}}{\alpha_1E_c}\right)+\left(\frac{4DWq^2\alpha_1\mu_0\varepsilon(x)E_c}{2Dq^2(d_{AlGaN}+d_{AlN})+\varepsilon(x)}\right)\left(\frac{E_1-2E_c}{E_1E_c}\right)$$

$$\beta_2=\left(\frac{8DWq^2\alpha_1\mu_0\varepsilon(x)E_c}{2Dq^2(d_{AlGaN}+d_{AlN})+\varepsilon(x)}\right)\left(\left(V_g-V_{th}(x)-\frac{k_bT}{q}\right)\left(\frac{E_c-E_1}{E_1E_c}\right)-L\right)-\left(\frac{4DWq^2\alpha_1\mu_0\varepsilon(x)E_c}{2Dq^2(d_{AlGaN}+d_{AlN})+\varepsilon(x)}\right)^2\left(\frac{R_{source}+R_{drain}}{\alpha_1E_c}+\left(2+\frac{R_{source}+2R_{drain}}{\alpha_1E_c}\right)\left(V_g-V_{th}(x)-\frac{k_bT}{q}\right)\right)$$

$$\beta_3 = \left(\left(\frac{4DWq^2\alpha_1\mu_0\varepsilon(x)E_c}{2Dq^2(d_{AlGaN}+d_{AlN})+\varepsilon(x)}\right)\left(V_g - V_{th}(x) - \frac{k_bT}{q}\right)\right)^2\left(1+\frac{R_{source}+2R_{drain}}{\alpha_1E_c}\right) + \left(\frac{8DLWq^2\alpha_1\mu_0\varepsilon(x)E_c}{2Dq^2(d_{AlGaN}+d_{AlN})+\varepsilon(x)}\right) \tag{15}$$

The HEMT is modelled according to the following specifications given in Table T1.

Table S1. Specifications of the

Parameters	Values
Gate Length	20μm
Gate Width	40μm
d_{AlGaN}	20nm
d_{GaN}	30 μm
N_D	10^{18}cm^{-3}
2DEG Hall Mobility	1800cm^2/(V-s)
Temperature	300K

For practical purposes, we have purposefully avoided including any scattering effects in HEMT. As we have stated earlier also that we intend to operate our HEMT at lowest field – highest mobility conditions, we have given special attention to accurately calculate the low field I_{ds}. This modelling helps us to affirm our concept, that with the highly mobile 2DEG, we can work in the linear region to couple it with the incumbent THz radiation and measure the residual V_{ds} and I_{ds}. The V_{ds} vs I_{ds} graph for various gate voltages is shown in figure S2.

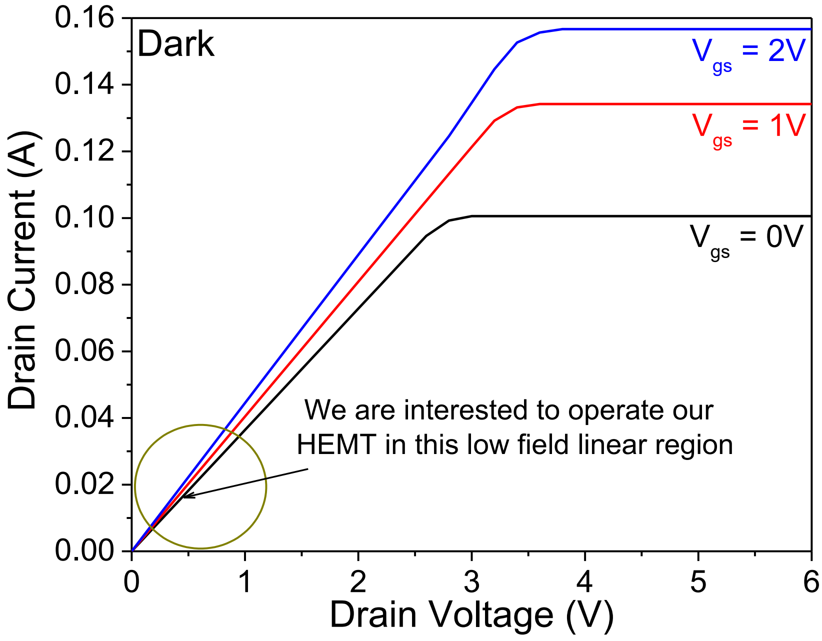

Figure S2. Drain voltage (V_{ds}) vs drain current (I_{ds}) graph for various gate voltages (without illumination).

2: Drude modelling of monolayer graphene

A quantity that determines the gain at frequency ω is the real part of the net dynamic conductivity Re σ_ω consisting of the intraband Re σ_ω^{intra} and the interband Re σ_ω^{inter} contributions; the negative values of Re σ_ω give the gain. Re σ_ω is proportional to the absorption of photons with frequency ω and given by[4],

$$Re\sigma_\omega = Re\sigma_\omega^{inter} + Re\sigma_\omega^{intra} = \frac{q^2\pi}{2h}(1-2f) + \frac{\left(ln2 + {E_F}/{2k_bT}\right)q^2\pi}{2h}\frac{2\pi k_bT}{h(1+\omega^2\tau^2)} \tag{16}$$

where h is the Planks constant, k_b is the Boltzmann constant, and τ is the momentum relaxation time of carriers. The intraband contribution Re σ_ω^{intra} corresponds to the Drude-like absorption and is always positive contributing a loss. Typical simulated results for Re σ_ω (absolute values) are shown in figure S3 as functions of time and frequency with momentum relaxation time of 10ps, after impulsive pumping with a photon energy of 0.8 eV and an intensity of 10^8 W/cm^2.

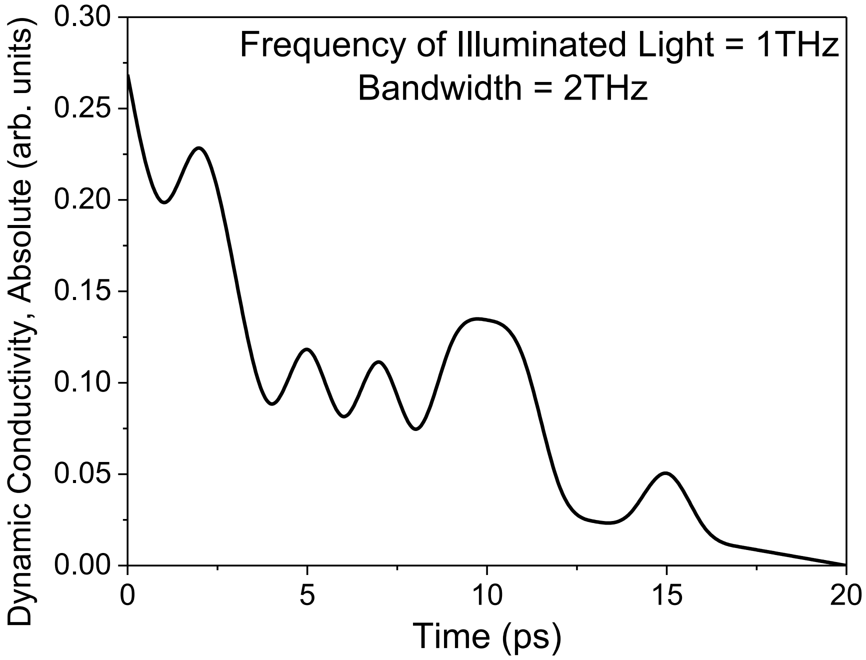

Figure S3. Graph depicting the time evolution of dynamic conductivity of graphene.

Thus far, all the experimental studies confirmed the following theoretical expectations. First, interband transitions dominate in the infrared/visible range. Second, intraband transitions dominate in the terahertz range, resulting in an optical conductivity well described by the Drude model. Graphene supports a high carrier concentration (up to 1×10^{14} cm^{-2}) as well as outstanding carrier mobility (> 20,000 cm^{2}V^{-1}s^{-1} for n_s ~5×10^{12} cm^{-2}) for both holes and electrons owing to its symmetric conical band structure, which leads to a good tunable conductivity range and a high maximum hole/electron conductivity comparable to that of conventional epitaxial semiconductors achievable only by electrons.

3: Working principle with 3D illustration of our THz detector

THz radiation is illuminating monolayer graphene which is a Drude-absorber, creating wavelength-limited carrier density oscillations which adds up as a small-signal ac component to the gate bias already applied, without suffering nearly any phonon losses due to single-atomic-layer structure of the graphene. The carrier density perturbations thus created are capacity-coupled to the two-dimensional electron gas (2DEG) if the highly mobile AlGaN/GaN HEMT on Si. The combination of capacity-coupled graphene plasmons excited by THz and 2DEG in the AlGaN/GaN HEMT is generating a substantial rectified voltage (ΔU), at room temperature with fast response times. The above simulated model is tailored according to the dimensions and gate voltages given in figure S4 which is taken from Table S1 of the analytical modelling. EM wave physics simulator (Sentaurus TCAD) is used for the virtual THz photogeneration on the detector. Operation of the detector under fully depleted conditions ensures a steady photogenerated voltage. The transistor characteristics (I_{ds}-V_{ds}) at V_{gs}=-3.45V shows the direct effect of THz rectification with increased potential asymmetry under depleted conditions. For the simulation of graphene, we have avoided modelling of the ballistic transport mechanism. We have used a carbon layer thinned down to a single atomic layer and fed all the related n, k values to the simulator. This is a good approximation for obtaining the Drude carrier modulations of graphene when exposed to THz radiation.

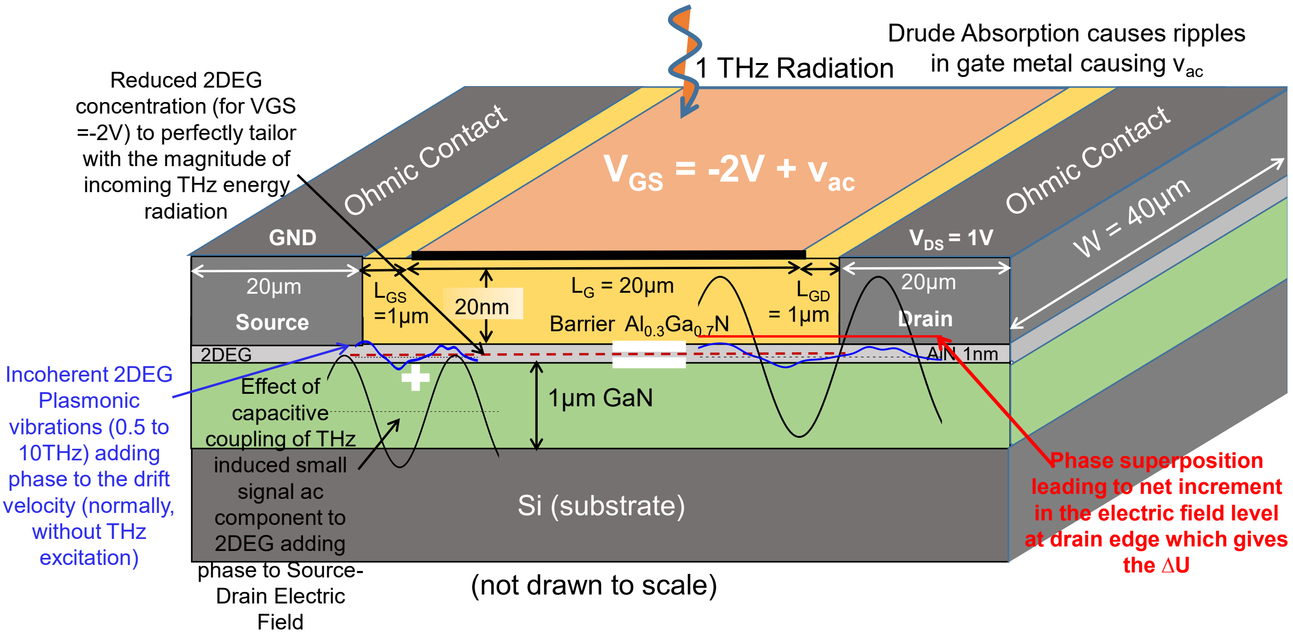

Figure S4. Schematic representation of the working of the graphene on AlGaN/GaN HEMT THz detector upon exposure to THz radiation.

4: Comparative analysis table of detector parameters

We give a more generalized comparative analysis of the detector parameters of different genres THz detector technologies with respect to our proposed technology is given in table T2. Our Graphene on AlGaN/GaN HEMT THz detector model has one of the highest responsivities compared to most of the bolometric and pyroelectric detectors. Noise Equivalent Power (NEP) of our proposed detector can also be

compared to many other RT and cryogenic transistor-based THz detectors as is evident from table T2. Since the parameters for our model are calculated from a simulation-based model, upon fabrication, the detector parameters might degrade slightly.

Table S2. Comparative chart for different THz detectors investigated till date and their detector parameters.

THz Detector Type	Operating Temperature (K)	Operating Frequency (THz)	Responsivity (V/W)	Response Time (s)	Noise Equivalent Power ($W/Hz^{1/2}$)
Bolometric Detectors[5]	< 4.2	0.1 to 30	10^7 to 10^5	10^{-2} to 10^{-3}	10^{-16} to 10^{-13}
High Temperature Pyroelectric Detectors[6]	240 to 350	0.1 to 30	10^5	10^{-2}	10^{-9}
Si FETs/CMOS[7]	295	0.2 to 4.3	30	10^{-9}	4.8×10^{-10}
Resonance with 2DEG Plasmons[8]	10	1.8 to 3.1	-	10^{-9}	-
Non-Resonant 2DEG Plasmons[9]	300	0.5 to 2.5	2.2×10^3	10^{-9}	1.5×10^{-11}
Bias Dependent THz Detection[10]	300	0.5 to 1	2×10^2	10^{-9}	10^{-10}
HEMTs with varied Gate Architecture[11]	300	0.1 to 1	1.5×10^4	10^{-9}	5×10^{-12}
HEMT with nano-antenna gate[12]	300	0.14	2.7×10^4	10^{-9}	10×10^{-12}
HEMT with asymmetric dual grating gate[4]	300	1	2.2×10^4	10^{-9}	15×10^{-12}
**Graphene on AlGaN/GaN HEMT (Our Model)**	**300**	**1**	**2.1×10^6**	**10^{-9} estimated**	**4.6×10^{-12}**

5: Comparative analysis table of similar dimension detectors

In table T3 we give a detailed comparative analysis of operating frequency, responsivity, and active area dimensions of those previous works whose dimensions are in micrometer-range similar to ours.

Table S3. Comparative chart for different THz detectors of similar dimensions compared to ours.

THz Detector Type	Operating Frequency (THz)	Responsivity (V/W)	Active Area Dimension (µm)
**Graphene on AlGaN/GaN HEMT (Our Model)**	**1**	**2.1×10^6**	**20 X 40**
Asymmetric dual grating gate HEMT[13]	0.1 to 4	10^3 @ 1 THz	20 X 20
Floating antenna GaN HEMT detector[14]	0.8 to 1	3.6×10^3	2 X 8
GaN HEMT Terahertz Detection	1 to 10	10^4	2 (gate length)

Model[15]			
THz FET Direct Detectors Based on CVD Graphene[16]	0.6	14	2 X 2.5

References

1. Muravjov, A. V; Veksler, D.B.; Popov, V. V; Polischuk, O. V; Pala, N.; Hu, X.; Gaska, R.; Saxena, H.; Peale, R.E.; Shur, M.S. Temperature Dependence of Plasmonic Terahertz Absorption in Grating-Gate Gallium-Nitride Transistor Structures. *Appl. Phys. Lett.* **2010**, *96*, 42105, doi:10.1063/1.3292019.

2. Nishimura, T.; Magome, N.; Khmyrova, I.; Suemitsu, T.; Knap, W.; Otsuji, T. Analysis of Fringing Effect on Resonant Plasma Frequency in Plasma Wave Devices. *Jpn. J. Appl. Phys.* **2009**, *48*, 04C096, doi:10.1143/JJAP.48.04C096.

3. Khan, M.S.I. Analytical Surface Charge Control Model for AlN/GaN/AlGaN Double Heterojunction Field-Effect Transistor. *J. Electr. Electron. Eng.* **2013**, *1*, 114–122, doi:10.11648/j.jeee.20130105.12.

4. Otsuji, T.; Watanabe, T.; Tombet, S.A.B.; Satou, A.; Knap, W.M.; Popov, V. V; Ryzhii, M.; Ryzhii, V. Emission and Detection of Terahertz Radiation Using Two-Dimensional Electrons in III–V Semiconductors and Graphene. *IEEE Trans. Terahertz Sci. Technol.* **2013**, *3*, 63–71, doi:10.1109/TTHZ.2012.2235911.

5. Knap, W.; Dyakonov, M.; Coquillat, D.; Teppe, F.; Dyakonova, N.; Łusakowski, J.; Karpierz, K.; Sakowicz, M.; Valusis, G.; Seliuta, D.; et al. Field Effect Transistors for Terahertz Detection: Physics and First Imaging Applications. *J. Infrared, Millimeter, Terahertz Waves* **2009**, *30*, 1319–1337, doi:10.1007/s10762-009-9564-9.

6. Watanabe, T.; Tombet, S.B.; Tanimoto, Y.; Wang, Y.; Minamide, H.; Ito, H.; Fateev, D.; Popov, V.; Coquillat, D.; Knap, W.; et al. Ultrahigh Sensitive Plasmonic Terahertz Detector Based on an Asymmetric Dual-Grating Gate HEMT Structure. *Solid. State. Electron.* **2012**, *78*, 109–114, doi:https://doi.org/10.1016/j.sse.2012.05.047.

7. Teppe, F.; Knap, W.; Veksler, D.; Shur, M.S.; Dmitriev, A.P.; Kachorovskii, V.Y.; Rumyantsev, S. Room-Temperature Plasma Waves Resonant Detection of Sub-Terahertz Radiation by Nanometer Field-Effect Transistor. *Appl. Phys. Lett.* **2005**, *87*, 52107, doi:10.1063/1.2005394.

8. Knap, W.; Teppe, F.; Dyakonova, N.; Coquillat, D.; Łusakowski, J. Plasma Wave Oscillations in Nanometer Field Effect Transistors for Terahertz Detection and Emission. *J. Phys. Condens. Matter* **2008**, *20*, 384205, doi:10.1088/0953-8984/20/38/384205.

9. Tanigawa, T.; Onishi, T.; Imafuji, O.; Takigawa, S.; Otsuji, T. AlGaN/GaN Plasmon-Resonant Terahertz Detectors with On-Chip Patch Antennas. In Proceedings of the Conference on Lasers and Electro-Optics/International Quantum Electronics Conference; Optica Publishing Group: Baltimore, Maryland, 2009; p. CThFF7.

10. Hou, H.; Liu, Z.; Teng, J.; Palacios, T.; Chua, S.-J. A Sub-Terahertz Broadband Detector Based on a GaN High-Electron-Mobility Transistor with Nanoantennas. *Appl. Phys. Express* **2017**, *10*, 14101, doi:10.7567/APEX.10.014101.

11. Otsuji, T.; Shur, M. Terahertz Plasmonics: Good Results and Great Expectations. *IEEE Microw. Mag.* **2014**, *15*, 43–50, doi:10.1109/MMM.2014.2355712.

12. Dyakonov, M.; Shur, M. Detection, Mixing, and Frequency Multiplication of Terahertz Radiation by Two-Dimensional Electronic Fluid. *IEEE Trans. Electron Devices* **1996**, *43*, 380–387, doi:10.1109/16.485650.

13. Kurita, Y.; Ducournau, G.; Coquillat, D.; Satou, A.; Kobayashi, K.; Boubanga Tombet, S.; Meziani, Y.M.; Popov, V. V; Knap, W.; Suemitsu, T.; et al. Ultrahigh Sensitive Sub-Terahertz Detection by InP-Based Asymmetric Dual-Grating-Gate High-Electron-Mobility Transistors and Their Broadband Characteristics. *Appl. Phys. Lett.* **2014**, *104*, 251114, doi:10.1063/1.4885499.

14. Sun, J.D.; Sun, Y.F.; Wu, D.M; Cai, Y.; Qin, H.; Zhang, B.S. High-Responsivity, Low-Noise, Room-Temperature, Self-Mixing Terahertz Detector Realized Using Floating Antennas on a GaN-Based Field-Effect Transistor. *Appl. Phys. Lett.* **2012**, *100*, 13506, doi:10.1063/1.3673617.

15. Meng, Q.; Lin, Q.; Wang, Z.; Wang, Y.; Jing, W.; Xian, D.; Zhao, N.; Yao, K.; Zhang, F.; Tian, B.; et al. Numerical Investigation of GaN HEMT Terahertz Detection Model Considering Multiple Scattering Mechanisms. *Nanomaterials* 2023, *13*, 632.

16. Zak, A.; Andersson, M.A.; Bauer, M.; Matukas, J.; Lisauskas, A.; Roskos, H.G.; Stake, J. Antenna-Integrated 0.6 THz FET Direct Detectors Based on CVD Graphene. *Nano Lett.* **2014**, *14*, 5834–5838, doi:10.1021/nl5027309.
